# Clinical Utility of Tau Positron Emission Tomography in the Diagnostic Workup of Patients With Cognitive Symptoms

**DOI:** 10.1001/jamaneurol.2023.1323

**Published:** 2023-05-22

**Authors:** Ruben Smith, Douglas Hägerström, Daria Pawlik, Gregory Klein, Jonas Jögi, Tomas Ohlsson, Erik Stomrud, Oskar Hansson

**Affiliations:** 1 Clinical Memory Research Unit, Department of Clinical Sciences, Lund University, Malmö, Sweden; 2 Department of Neurology, Skåne University Hospital, Lund, Sweden; 3 Department of Neurophysiology, Skåne University Hospital, Lund, Sweden; 4 F. Hoffmann-La Roche Ltd, Basel, Switzerland; 5 Skåne University Hospital, Department of Clinical Physiology and Nuclear Medicine, Lund, Sweden; 6 Department of Radiation Physics, Skåne University Hospital, Lund, Sweden; 7 Memory Clinic, Skåne University Hospital, Malmö, Sweden

## Abstract

**Importance:**

It is important to determine the added clinical value for tau positron emission tomography (PET) in the diagnostic workup of patients with cognitive symptoms before widespread implementation in clinical practice.

**Objective:**

To prospectively study the added clinical value of PET detecting tau pathology in Alzheimer disease (AD).

**Design, Setting, and Participants:**

This prospective cohort study (Swedish BioFINDER-2 study) took place from May 2017 through September 2021. A total of 878 patients with cognitive complaints were referred to secondary memory clinics in southern Sweden and then recruited to the study. In total, 1269 consecutive participants were approached, but 391 did not meet inclusion criteria or did not complete the study.

**Exposures:**

Participants underwent a baseline diagnostic workup, including clinical examination, medical history, cognitive testing, blood and cerebrospinal fluid sampling, magnetic resonance imaging of the brain, and a tau PET ([^18^F]RO948) scan.

**Main Outcomes and Measures:**

The primary end points were change in diagnosis and change in AD drug therapy or other drug treatment between the pre- and post-PET visits. A secondary end point was the change in diagnostic certainty between the pre- and post-PET visits.

**Results:**

A total of 878 participants with a mean age of 71.0 (SD, 8.5) years (491 male [56%]) were included. The tau PET result led to a change in diagnoses in 66 participants (7.5%) and a change in medication in 48 participants (5.5%). The study team found an association with overall increased diagnostic certainty after tau PET in the whole data set (from 6.9 [SD, 2.3] to 7.4 [SD, 2.4]; *P* < .001). The certainty was higher in participants with a pre-PET diagnosis of AD (from 7.6 [SD, 1.7] to 8.2 [SD, 2.0]; *P* < .001) and increased even further in participants with a tau PET positive result supporting an AD diagnosis (from 8.0 [SD, 1.4] to 9.0 [SD, 0.9]; *P* < .001). The association with tau PET results had the largest effect sizes in participants with pathological amyloid-β (Aβ) status, whereas no significant change in diagnoses was seen in participants with normal Aβ status.

**Conclusions and Relevance:**

The study team reported a significant change in diagnoses and patient medication when tau PET was added to an already extensive diagnostic workup that included cerebrospinal fluid AD biomarkers. Including tau PET was associated with a significant increase in certainty of underlying etiology. The effect sizes for certainty of etiology and diagnosis were largest in the Aβ-positive group and the study team suggests that clinical use of tau PET be limited to populations with biomarkers indicating Aβ positivity.

## Key Points

**Question:**

Does tau positron emission tomography (PET) provide additional information on top of an extensive clinical workup in participants with cognitive symptoms?

**Findings:**

In this cohort study of 878 patients referred to secondary memory clinics in south Sweden, the study team found that including tau PET in the diagnostic workup resulted in a statistically significant change in diagnosis in 7.5% of the participants and a significant change in medication in 5.5% of the study population and also found a significant association of overall increased diagnostic certainty with including tau PET.

**Meaning:**

Tau PET may have an added clinical value to increase diagnostic certainty, especially in amyloid-β positive patients where Alzheimer disease is a differential diagnosis.

## Teaser

This study aims to prospectively study the added clinical value of positron emission tomography detecting tau pathology in Alzheimer disease.

## Introduction

Alzheimer disease (AD) is believed to be caused by the accumulation of amyloid-β (Aβ) in the brain^1,2,3^ followed by a gradual spread of tau pathology across the brain as the clinical symptoms emerge.^4,5,6^ Aβ positron emission tomography (PET) for determining the presence of Aβ pathological changes in the brain has been available for more than a decade and there are now several tracers available for clinical use (eg, [^18^F]florbetapir, [^18^F]flutemetamol, and [^18^F]florbetaben). A large prospective multicenter study from the US (the IDEAS study)^7^ reported that the added information from Aβ-PET led to a significant change in patient management and diagnosis in patients where AD was among the considered differential diagnoses. A recent retrospective study^8^ showed that both Aβ-PET and tau PET increased diagnostic confidence and changed diagnoses to a similar extent when added to a basic clinical workup. There are now a number of tau PET tracers available for research purposes.^9^ The most widely used tracer, [^18^F]flortaucipir, has been shown to reliably detect tau as assessed by neuropathology^10,11,12,13^ and has been approved by the US Food and Drug Administration for use in the US as a diagnostic marker of neurofibrillary tau in AD.^14^ It is expected that tau PET will also be available for clinical use in other parts of the world within the coming years. The recently developed second-generation tau tracer [^18^F]RO948, used in the current study, has shown similar properties as flortaucipir.^15^ Before widespread implementation of tau PET as a diagnostic method in the clinic, it is vital to establish if the method shows an added clinical value for diagnosis and treatment of patients with memory complaints and to establish whether the method increases the diagnostic confidence of treating clinicians.

The aim of the current study was to prospectively evaluate the added clinical value of including visual read of tau PET ([^18^F]RO948 PET) in the diagnostic workup of AD. This was evaluated in secondary memory clinics where the diagnostic workup already included patient history, clinical examination, cognitive testing, magnetic resonance imaging (MRI) of the brain, as well as relevant cerebrospinal fluid (CSF) markers (Aβ42, Aβ40, and pTau181). Specifically, we studied whether tau PET led to change in diagnoses, change in treatment, as well as in diagnostic certainty. As tau PET examinations are costly, we have also assessed whether this biomarker is more informative in certain subpopulations.

## Methods

### Participants and Baseline Assessment

Between May 2017 and Sept 2021, 1269 patients referred for cognitive or neurological symptoms were consecutively recruited at the secondary memory clinic at Skåne University Hospital, Malmö, Sweden; the secondary memory clinic at Ängelholm hospital, Ängelholm, Sweden; and the secondary neurology clinic, Skåne University Hospital, Lund, Sweden. The study was part of the BioFINDER-2 study (NCT03174938). Inclusion and exclusion criteria have been specified in detail previously.^16^ Patients included in this study had either subjective cognitive decline (SCD) or objective reductions in memory performance, either at mild cognitive impairment (MCI) level or dementia level. A total of 391 participants were excluded (eMethods in Supplement 1 for details) and 878 participants met the inclusion criteria and completed the study. Participant characteristics are presented in the Table and the eTable in Supplement 1. Written informed consent was obtained from all participants prior to entering the study. The study was approved by the regional review board for human research ethics at Lund University. Strengthening the Reporting of Observational Studies in Epidemiology (STROBE) reporting guidelines were followed.

**Table.** Participant Demographics

Characteristic	AD	Non-AD	AD-SCD	AD-MCI	AD-dementia	*P* value
No.	408	470	33	179	196	NA
Age, y, mean (SD)	72.7 (7.8)^a^	69.5 (8.9)	70.4 (8.4)^b^	72.2 (7.6)	73.5 (7.9)	^a^ ^,^ ^b^
Sex						
Female	192	195	11	79	102	.11
Male	216	275	22	100	94	.11
MMSE score, mean (SD)	24.4 (4.6)^a^	26.2 (4.2)	29.1 (1.1)^c^	26.7 (2.5)^d^	21.4 (4.4)	^a^ ^,^ ^c^ ^,^ ^d^
Education, y, mean (SD)	12.6 (4.2)	12.3 (3.8)	14.4 (4.0)^e^	12.5 (4.3)	12.4 (4.2)	^e^
Aβ+, No./total No. (%)	386/394 (98)^a^	167/454 (37)	32/32 (100)	169/172 (98)	185/190 (97)	^a^
Positive tau PET, No./total No. (%)^f^	304/408 (75)^a^	39/470 (8)	11/33 (33)^g^	122/179 (68)^d^	171/196 (87)	^a^ ^,^ ^g^ ^,^ ^d^
Tau PET SUVR, temporal ROI, mean (SD)	1.77 (0.64)^a^	1.19 (0.20)	1.28 (0.25)^c^	1.59 (0.49)^d^	2.02 (0.70)	^a^ ^,^ ^c^ ^,^ ^d^

Abbreviations: Aβ+, amyloid-β positive; AD, Alzheimer disease; MCI, mild cognitive impairment; MMSE, Mini-Mental State Examination; Non-AD, all conditions other than AD pooled (eMethods in Supplement 1); NA, not applicable; PET, positron emission tomography; ROI, region of interest; SCD, subjective cognitive decline; SUVR, standardized uptake value ratio.

^a^ *P* < .001 vs non-AD.

^b^ *P* = .04 vs AD with dementia.

^c^ *P* < .001 vs AD with MCI and AD with dementia.

^d^ *P* < .001 vs AD with dementia.

^e^ *P* = .01 vs AD with MCI and AD with dementia.

^f^ Based on visual read.

^g^ *P* < .001 vs AD with MCI and *P* < .001 AD with dementia.

**Figure 1.**
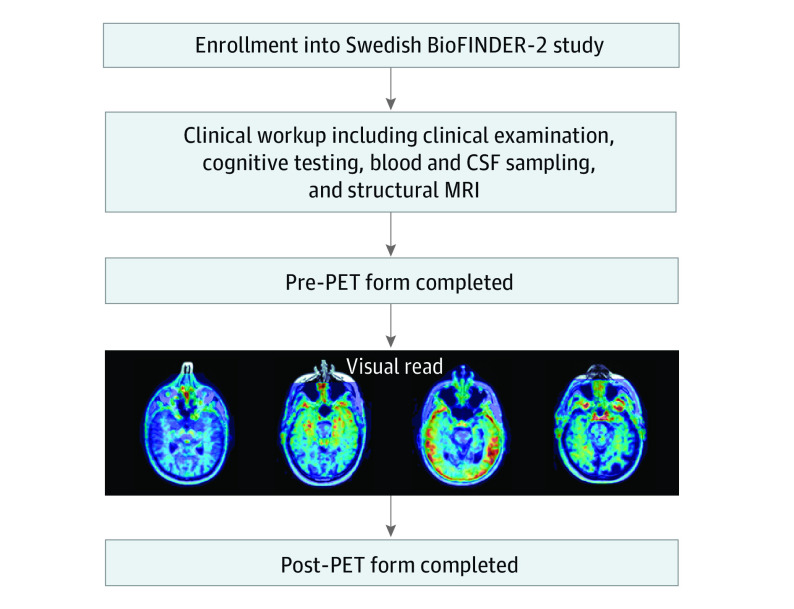
Study Design Patients with memory complaints were recruited to the study after a comprehensive clinical workup, including clinical examination, cognitive testing, structural imaging using magnetic resonance imaging (MRI), and blood/cerebrospinal fluid (CSF) sampling. The clinician filled out the pre–positron emission tomography (PET) form, indicating diagnosis, suspected underlying etiology, certainty of etiology, and treatment. The clinician then received the outcome of the visual read and filled out the post-PET form.

The study design is presented in Figure 1. Participants were assessed with a baseline diagnostic workup, including clinical examination, medical history, cognitive testing, CSF sampling for biomarkers (mainly Aβ42, Aβ40, and pTau181), and MRI imaging. Based on the baseline assessment, the treating clinician was asked to fill out a report form stating (1) the most likely diagnosis (etiology) underlying the cognitive symptoms, (2) how certain they were of this diagnosis on a scale from 0 (very uncertain) to 10 (very certain), (3) the cognitive status (SCD/MCI/dementia) of the participant, (4) the certainty of the cognitive status (scale 0 to 10), (5) if the patient was receiving any medication to enhance cognitive function (for example, acetylcholinesterase inhibitors or memantine) or antidepressants, and (6) if any further investigations were planned for establishing the diagnosis. Once the form was filled out, the visual read of the [^18^F]RO948 PET was revealed together with a template for interpretation (eAppendix in Supplement 1) and the clinician was asked to fill out a follow-up report form, again stating the most likely etiology, their certainty, as well as if they planned changes in the medication regimen or patient management as a result of the [^18^F]RO948 PET information. The full forms, translated into English, are available in the eAppendix in Supplement 1. Outcomes were change in diagnosis (from AD to non-AD or vice versa), change in medication to enhance cognitive function or antidepressant medication, and change in diagnostic certainty.

### Image Acquisition and Processing

Imaging acquisition details are provided in the eMethods in Supplement 1. Rating was performed by 2 raters (D.H. and R.S.), masked to clinical information of the patient and reaching a joint decision on which category to allot the image. In brief, the categories used were (A) normal image; no discernible [^18^F]RO948 retention, (B) retention of [^18^F]RO948 confined to the temporal lobes, (C) more widespread retention of [^18^F]RO948, reaching into the parietal, occipital, or frontal lobes, and (D) inconclusive scan. A detailed description of the visual read algorithm and example images are provided in the eMethods and eFigure 1 in Supplement 1. For analyses using a dichotomous negative/positive tau PET read, inconclusive visual reads were considered negative (not having an AD-typical pattern).

The visual read algorithm applied in this study was relatively similar to the visual read algorithm used by Seibyl et al^17^ for assessing [^18^F]MK-6240 PET scans, but allowed a positive visual read of the tau PET scan even with unilateral uptake in the medial temporal lobe. The visual read algorithm used in this study is also likely to be more sensitive to early temporal accumulation of tau compared with published methods for [^18^F]flortaucipir.^8,10^

### Statistics

Statistical comparisons of baseline characteristics were assessed using χ^2^ test for categorical data (sex) or Mann-Whitney *U* tests for continuous data. For comparison of pre- and post-tau PET, paired Wilcoxon signed rank tests or the McNemar χ^2^ tests with continuity correction were used. For comparison between nonpaired groups, Wilcoxon rank sum tests were performed. All statistical tests were 2-sided with a significance level of .05. All analyses were performed using the R programming language version 4.0.3 (The R Project).

## Results

### Participants

Patients (n = 878) from the 3 secondary clinics were consecutively recruited into the BioFINDER-2 study. Participant demographics are summarized in the Table. Based on the pre-PET forms, 408 participants had an AD diagnosis and 470 had non-AD diagnoses (details in the eMethods and the eTable in Supplement 1). Patients with a pre-PET diagnosis of AD were older and performed worse on Mini-Mental State Examination compared with the non-AD group. Of the participants with a pre-PET diagnosis of AD, 304 of 408 (75%) had tau PET scans that were read as positive (ie, read as early or late AD pattern). Furthermore, 11 of 33 patients with SCD, where AD was suspected as an underlying cause before PET (33%), had a positive tau PET scan; 122 of 179 patients with MCI, where AD was suspected as underlying cause before PET (68%), had a positive tau PET scan; and 171 of 196 patients with dementia, where AD was suspected as underlying cause before PET (87%), had a positive tau PET scan. In the group with a non-AD diagnosis before PET, 39 of 470 had positive tau PET scan (8%) (eFigure 2 in Supplement 1).

### Association With Tau PET on Diagnosis and Diagnostic Certainty

**Figure 2.**
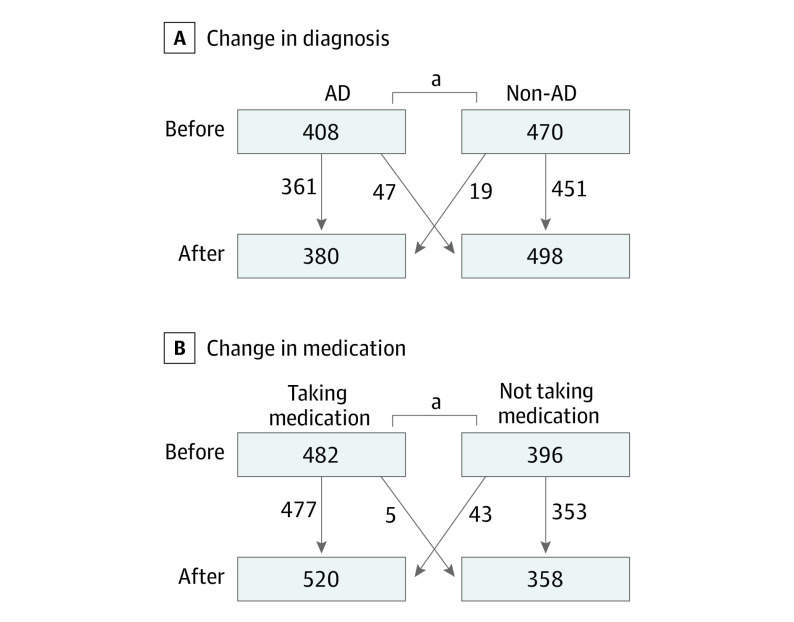
Changes in Diagnoses and Medication A, McNemar test, *P* < .001. B, McNemar, test *P* < .001. Participants with an increase in medication (on top of preexisting cognitive medication) are considered as being off of medication at baseline. AD indicates Alzheimer disease.

The tau PET information was associated with a change in diagnoses in 66 out of 878 participants (7.5%). Diagnoses changed from AD to non-AD in 47 participants (11.5% of participants with a pre-PET diagnosis of AD) and from non-AD to AD in 19 participants (4.0% of participants with a non-AD pre-PET diagnosis) (McNemar test,  *P* < .001; Figure 2A). Changes in diagnoses were found both in patients with a negative tau PET visual read (47 changed diagnosis, total n = 535; McNemar test, *P* < .001), as well as in patients within the group with a positive visual read (19 changed diagnosis, total n = 343; McNemar test, *P* = .001). Significant changes in diagnoses were seen in participants with dementia (23 changed diagnosis, total n = 318; McNemar test, *P* = .04) and MCI (33 changed diagnosis, total n = 419; McNemar test, *P* = .01), whereas the number of changed diagnoses in the SCD group did not reach statistical significance (10 changed diagnosis, total n = 141; McNemar test, *P* = .75; eFigure 3 in Supplement 1).

Eight hundred forty-eight of the participants (97%) had information on their Aβ status determined using the CSF Aβ42/40 ratio. Therefore, the study team studied the added value of tau PET in Aβ-positive (n = 553) and Aβ-negative (n = 295) subpopulations. In the Aβ-positive group, the tau PET result led to a significant change in diagnoses in 55 participants (9.9%) (38 changed from pre-PET AD to a post-PET non-AD diagnosis and 17 from a pre-PET non-AD to a post-PET AD diagnosis; McNemar test, *P* = .01). The visual read results of the Aβ-positive group are presented in eFigure 4 in Supplement 1. The tau PET result was not associated with a change in diagnoses in the Aβ-negative group (6 participants changed from pre-PET AD to post-PET non-AD and 1 from a pre-PET non-AD diagnosis to post-PET AD; McNemar test, *P* = .13).

**Figure 3.**
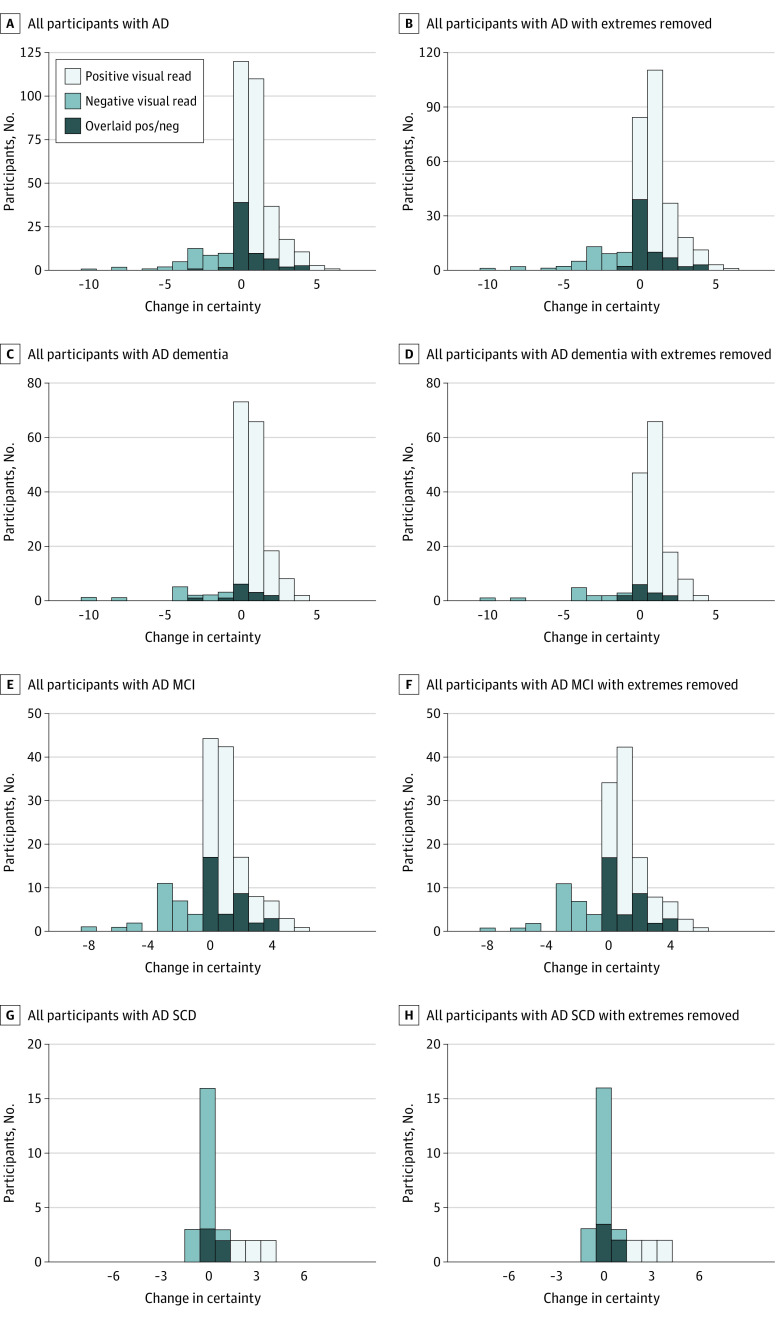
Change in Certainty in Participants With a Pre–Positron Emission Tomography (PET) Alzheimer Disease (AD) Diagnosis Change was calculated as certainty post-PET minus certainty pre-PET. The results from subgroups with different levels of cognitive impairment are shown in C, dementia; E, mild cognitive impairment (MCI); and G, subjective cognitive decline; (SCD). Since participants beginning at certainty level 10 cannot increase and 0 cannot decrease, results where individuals starting at 10 (for positive visual reads) or 0 (for negative visual reads) have been removed and resulting graphs are shown for all participants in B; and participants with D, dementia; F, MCI; and H, SCD.

In the total population, the overall certainty of diagnosis increased from 6.9 (SD, 2.3) to 7.4 (SD, 2.4) (*P* < .001) on a scale from 0 (very uncertain) to 10 (complete certainty). The certainty was higher in the group having a pre-PET diagnosis of AD (n = 408; Figure 3A and 3B), increasing from 7.6 (SD, 1.7) to 8.2 (SD, 2.0) (*P* < .001), and among the patients with a pre-PET AD diagnosis having a positive tau PET scan (supportive of an AD diagnosis) where certainty increased from 8.0 (SD, 1.4) to 9.0 (SD, 0.9) (*P* < .001). In patients with a pre-PET AD diagnosis, having a negative tau PET scan visual dropped read certainty from 6.5 (SD, 2.0) to 5.7 (SD, 2.1) (*P* < .001). The increase in certainty in patients with a pre-PET AD diagnosis was present in participants irrespective of cognitive status: AD dementia (n = 196; 8.2 [SD, 1.3] vs 8.7 [SD, 1.7]; *P* < .001 [Figure 3C and 3D]), MCI (n = 179; 7.3 [SD, 1.8] vs 7.8 [SD, 2.1]; *P* < .001 [Figure 3E and 3F]), and SCD (n = 33; 6.2 [SD, 1.7] vs 6.8 [SD, 1.8]; *P* = .01 [Figure 3G and 3H]). Since pre-PET rating at the highest and lowest certainty levels (ie, 0 and 10) can either not increase or decrease in certainty, the study team presented the same data where these values have been removed (Figure 3B-3H). Graphs showing paired data of the changes in certainty in the AD diagnostic group are provided in eFigure 5 in Supplement 1.

The diagnostic certainty after the tau PET scan was lower in participants with a pre-PET non-AD diagnosis compared with a pre-PET AD diagnosis (6.7 [SD, 2.6] vs 8.2 [SD, 2.0]; *P* < .001), but there was still a significant, yet subtle, increase in certainty in the non-AD group after receiving the tau PET result (6.3 [SD, 2.5] vs 6.7 [SD, 2.6]; *P* < .001; eFigure 6 in Supplement 1).

In the Aβ-positive group, the tau PET result was associated with increased overall certainty of diagnoses from 7.2 (SD, 2.0) to 7.8 (SD, 2.2) (*P* < .001). Within the Aβ-positive group, significant increases in certainty were seen irrespective of cognitive status (SCD, 6.0 [SD, 2.0] to 6.5 [SD, 2.3]; *P* = .01; MCI, 6.8 [SD, 2.1] to 7.4 [SD, 2.3]; *P* < .001; and dementia, 7.9 [SD, 1.6] to 8.5 [SD, 1.7]; *P* < .001). There was a significant change of certainty in diagnosis also in the Ab-negative group, but the change was smaller in magnitude (from 6.5 [SD, 2.5] to 6.7 [SD, 2.6]; *P* < .001).

### Association of Tau PET With Treatment

At baseline, 506 of all 878 participants were receiving antidepressant medication or medication to enhance cognitive functions (eg, acetylcholinesterase inhibitors or memantine), or had tried medication but discontinued due to adverse effects or lack of effect. A total of 308 of 408 patients with a pre-PET AD diagnosis (75%) were receiving medication to enhance cognitive functions or had tried such a medication. This encompassed 185 of 196 patients with a pre-PET AD dementia diagnosis (94%), 118 of 179 with pre-PET MCI due to AD diagnosis (66%), and 5 of 33 with SCD where AD was judged as the underlying cause before PET (15%). In the total study population, 17 participants started receiving medication to enhance cognitive function and 21 had an addition of a medication with a different mechanism (acetylcholinesterase inhibitors added to memantine or the other way around) after receiving the tau PET result. For 5 patients, the tau PET result led to start or increase of antidepressant medication and in 5 participants the medication to enhance cognitive functions was discontinued (McNemar’s test, *P* < .001; Figure 2B). In total, changes in cognitive or antidepressant medication were made in 48 participants of the total population (5.5%). In a sensitivity analysis, only including changes in medication to enhance cognitive functions, the study team found similar results (eFigure 7 in Supplement 1).

## Discussion

The visual read of the tau PET led to a change in the diagnosis of 7.5% of the total population with 11.5% of those with a pre-PET AD diagnosis changing to a post-PET non-AD diagnosis and 4.0% of those with a pre-PET non-AD diagnosis changing to a post-PET AD diagnosis. Similarly, the result led to a significant change in medication with 48 participants receiving or discontinuing medication after the tau PET examination (5.5%). These numbers may seem small, but 75% of AD participants (including 92% with AD dementia) were already receiving medication at baseline.

We found that increased AD diagnosis certainty was associated with tau PET in participants where AD was the primary suspected pre-PET etiology, irrespective of cognitive status. Certainty decreased in the event of an unexpected negative scan in this patient population. The decrease was more marked in participants with an objective cognitive impairment (Figure 3C-F), whereas no change in certainty was seen with a negative scan in the SCD population with a suspected pre-PET AD etiology (Figure 3G and H). Ruling out underlying AD based on a negative tau PET result in the absence of clear objective cognitive impairment is questionable.^18,19^ This is likely the reason why no decrease in certainty was seen in the participants with SCD with suspected underlying AD etiology and a negative visual read. In line with this, a significant change in diagnoses was not seen in the SCD group, but only seen in the MCI and dementia groups.

The results of this study indicate an added value of including tau PET on top of an already extensive clinical workup, which included CSF AD biomarkers. CSF biomarkers are increasingly used in many memory clinics because of the high diagnostic accuracy and relatively low costs, and several CSF AD biomarker assays have recently been approved for clinical use by the US Food and Drug Administration.^20,21^ Still, lumbar puncture is invasive, but some novel blood-based biomarkers are reaching diagnostic accuracies that are similar to those of CSF AD biomarkers,^6,22^ which is why such blood markers will likely precede a decision to perform tau PET imaging in most memory clinics in the coming years. Therefore, the current prospective study will likely also be relevant to clinical settings where high-performing blood AD biomarkers will be used instead of CSF AD biomarkers.

The relatively large number of participants changing from a pre–PET AD to a post-PET non-AD underlying diagnosis in the present study suggests that relying solely on CSF biomarkers, such as Aβ42/Aβ40 or Aβ42/pTau, may result in an overestimation of the AD element in the etiology of the cognitive decline. These fluid biomarkers change early in the disease development and the biomarkers are pathological in a relatively high proportion of cognitively unimpaired individuals.^18,19^ Therefore, using fluid Aβ and pTau biomarkers alone may lead to difficulties to separate if subtle cognitive symptoms are derived from an early-stage AD or whether they are due to other causes, such as stress, depression, or another concomitant non-AD neurodegenerative disease.^19,23^ On the other hand, tau PET changes later in the AD disease course^24^ and is more closely associated with cognitive deterioration than fluid AD biomarkers.^6,25,26,27,28^

We found that including tau PET in the diagnostic workup in a secondary memory clinic setting provided best information when AD is the primary suspected pre-PET diagnosis. Similarly, in a subanalysis of CSF Aβ-positive and CSF Aβ-negative individuals, we found no clear added value of tau PET in the CSF Aβ-negative subgroup, but a clear change in the CSF Aβ-positive group. In the former group, there was a minor increase in the diagnostic certainty, but no association with change of diagnoses. We therefore suggest that tau PET should mainly be used in individuals where fluid biomarkers indicate presence of AD pathology.

It has been suggested that in case of cognitive impairment at the MCI or dementia level, the tau PET results might be used both to rule in and to rule out AD as the underlying primary pathology,^23^ but in cognitively unimpaired patients with SCD, the tau PET result might only be used to rule in AD pathology as the underlying cause.^16,25,29,30^ We recommend that tau PET is only used in patients with SCD when there is an increased risk of an underlying AD pathology and where the information may result in a beneficial change in patient management.

The association of Aβ PET with patient management and diagnosis has been studied previously in the large US multicenter IDEAS study.^7^ The authors found an association with patient management with 60.2% to 63.5% of study participants having a change in management as a result of the Aβ PET scan and diagnoses were changed in 25.1% and 10.5% with positive and negative Aβ PET scans, respectively. The largest effects on management were seen in patient medication where 43.6% to 44.9% of participants with a positive scan had a change in treatment regimen.^7^ In our study, the changes in treatment were more modest, probably reflecting that a large proportion of the patients participating were receiving medication already at baseline after having received results from fluid AD biomarkers in addition to the clinical assessments. However, our results indicate that tau PET change management, also when high-performing fluid AD biomarkers, has been used during the early stages of the diagnostic workup.

A previous retrospective study^8^ evaluated the added value of Aβ PET and tau PET for diagnosis and diagnostic certainty on top of a clinical workup, including clinical and neuropsychological assessment and MRI, but no blood or CSF biomarkers. The study included 136 memory clinic patients and found similar added values of Aβ and tau PET with an increase in diagnostic certainty and a similar degree of changed diagnoses (28% change) for the 2 PET methods.^8^ In our study, where fluid AD biomarkers were already known before tau PET imaging was performed, the change in diagnoses was as expected smaller (7.5%). This was comparable with the added diagnostic value of tau PET after having the Aβ PET result in the previous retrospective study (9%).^8^ The design of the previous study was very different from the design of our current study. The diagnoses and diagnostic certainty were determined based on summarized clinical information, cognitive tests, biomarkers, and imaging data in a retrospective fashion without seeing the patients.^8^ The advantage of the study design applied in this study is the prospective approach, a real-world memory clinic setting, and the larger study size. That said, both studied provided rather similar results. Does an increased diagnostic certainty and changed diagnoses matter for the patients? With emerging new treatments for AD being developed, an increased diagnostic certainty is becoming more important, but also in absence of a specific treatment a recent study has shown delayed institutionalization, lower mortality, and reduced care costs in participants receiving Aβ PET for diagnosis compared with participants not undergoing the PET scan.^31^

### Limitations

There are several limitations to the current study. First, all 3 secondary centers, including patients, are located in Sweden and the study population is ethnically rather homogenous; therefore generalizability to other ethnic groups and centers may be lower. Second, despite the cohort being large, the number of participants in some subgroups, such as SCD with a pre-PET suspected underlying AD, were relatively low and results will need to be interpreted with this in mind. Third, the study design did not include a control group. Fourth, even though most participants with positive visual reads were Aβ positive (98%), we cannot fully rule out that some early B reads might represent primary age-related tauopathy. Additionally, when the study started in 2017, high-performing blood-based biomarkers were not available in clinical practice, which is why CSF AD biomarkers were used instead before tau PET imaging.

## Conclusions

In this cohort study, we found that tau PET added value to an already extensive diagnostic workup, including high-performing fluid AD biomarkers. The tau PET results were associated with an increased diagnostic certainty and change in diagnoses and medication of the participants. The added value of tau PET was most pronounced in CSF Aβ-positive participants and we suggest that the clinical use of tau PET be limited to a population where fluid AD biomarkers are abnormal.

## Notes

Supplement 1. eMethods. Included/excluded participants, non-AD diagnoses, and imaging and visual read protocol eTable. Demographics of subcohorts eFigure 1. Visual read categories eFigure 2. Visual read results in different diagnostic and cognitive groups eFigure 3. Change in diagnoses in cognitive subgroups eFigure 4. Visual read results in amyloid-positive participants eFigure 5. Certainty in participants with AD diagnosis eFigure 6. Change in certainty in participants with a non-AD diagnosis at baseline eFigure 7. Changes in medication to enhance cognition eAppendix. Pre-PET and post-PET forms eReferences

Supplement 2. Data sharing statement
