# Supplementary material for: Clinical Utility of Tau Positron Emission Tomography in the Diagnostic Workup of Patients With Cognitive Symptoms
Source: JAMA Neurol. 2023 May 22;80(7):749–56. doi: 10.1001/jamaneurol.2023.1323 (PMC10203972; doi:10.1001/jamaneurol.2023.1323)
Supplement: Supplement 1. — eMethods. Included/excluded participants, non-AD diagnoses, and imaging and visual read protocol eTable. Demographics of subcohorts eFigure 1. Visual read categories eFigure 2. Visual read results in different diagnostic and cognitive groups eFigure 3. Change in diagnoses in cognitive subgroups eFigure 4. Visual read results in amyloid-positive participants eFigure 5. Certainty in participants with AD diagnosis eFigure 6. Change in certainty in participants with a non-AD diagnosis at baseline eFigure 7. Changes in medication to enhance cognition eAppendix. Pre-PET and post-PET forms eReferences [file jamaneurol-e231323-s001.pdf]

Supplemental Online Content

Smith R, Hägerström D, Pawlik D, et al. Clinical utility of tau positron emission tomography in the diagnostic workup of patients with cognitive symptoms. *JAMA Neurol*. Published online May 22, 2023. doi:10.1001/jamaneurol.2023.1323

eMethods. Included/excluded participants, non-AD diagnoses, and imaging and visual read protocol
eTable. Demographics of subcohorts
eFigure 1. Visual read categories
eFigure 2. Visual read results in different diagnostic and cognitive groups
eFigure 3. Change in diagnoses in cognitive subgroups
eFigure 4. Visual read results in amyloid-positive participants
eFigure 5. Certainty in participants with AD diagnosis
eFigure 6. Change in certainty in participants with a non-AD diagnosis at baseline
eFigure 7. Changes in medication to enhance cognition
eAppendix. Pre-PET and post-PET forms
eReferences

This supplemental material has been provided by the authors to give readers additional information about their work.

© 2023 Smith et al. *JAMA Neurology.*

eMethods

Included/excluded participants

In total 1269 patients referred for cognitive or neurological symptoms were consecutively recruited. 391 participants were excluded from analysis due to: withdrawn consent (n=202), did not undergo tau-PET scan (n=4), died prior to undergoing tau-PET (n=4), did not meet the inclusion criteria (n=140), or having missing data or ambiguously filled-out forms used for the evaluation of the study (n=41).

Non-AD diagnoses

Non-AD diagnoses included 100 participants with vascular dementia, 52 with frontotemporal dementia, 49 with dementia with Lewy bodies, 105 with other specified neurodegenerative disorders (such as Parkinson's disease, progressive supranuclear palsy *etc*), 128 with other, non-neurodegenerative, disorders underlying their cognitive decline (e.g. depression, stress, alcohol), and 36 where the diagnosis remained unclear after the initial work-up (eTable 1).

Imaging and Visual read protocol

We acquired [^{18}F]RO948 PET data, on digital GE Discovery MI PET/CT scanners. Image data was collected in the 70-90 min interval post injection of ~370 MBq radiolabeled [^{18}F]RO948. Low-dose CT scans were performed immediately prior to the PET scans for attenuation correction. PET data was attenuation corrected and reconstructed using the GE-developed Q.Clear algorithm (BSREM - Block sequential regularized expectation maximization, with beta = 150),[1] and 25.6-cm field of view (256 × 256 matrix). Images were visually assessed using a rainbow color scale with the reference cerebellar cortex set at the blue to cyan color shift. Images were co-registered to the low dose CT scans acquired for attenuation correction at the time of PET imaging. CT scans were used to provide some additional anatomical detail and to assess whether suspected off-target binding was localized to bone/meningeal structures. A visual read algorithm was applied where increased retention was determined in the 1) medial temporal lobes/entorhinal cortex and the temporal cortices, and 2) other neocortical regions. In cases where suspected non-AD related increases in [^{18}F]RO948 signal was detected, for example binding to meningiomas or due to stroke/hemorrhages, the binding was noted in the written response to the clinicians but

© 2023 Smith et al. *JAMA Neurology.*

indicated as a non-AD related pattern. Scans were assigned into one of four categories: A) Normal image, no discernible [^{18}F]RO948 retention; B) Retention of [^{18}F]RO948 confined to the temporal lobes; C) More widespread retention of [^{18}F]RO948, reaching into the parietal, occipital or frontal lobes; and D) Inconclusive scan, due to for example difficulties deciding whether a low level of binding in the medial temporal lobe represents true binding, off-target binding in the meninges close to the entorhinal cortex or other variations in the regional entorhinal uptake. In categories B-D a more detailed description of the uptake pattern was provided, indicating the location and extent of the binding (B and C) or a description of the reason why a scan was considered inconclusive (D). Unilateral medial temporal lobe uptake, if unequivocal, was considered being positive in the B category. Example images of the different categories are shown below in eFigure 1.

© 2023 Smith et al. *JAMA Neurology.*

eTable 1. Demographics of subcohorts

	Alzheimer's Disease (AD)	Vascular Dementia (VaD)	Fronto-temporal Dementia (FTD)	Dementia with Lewy Bodies (DLB)	Other specified Neuro-degenerative Disorder (ND)	Other, non-Neuro-degenerative disorder (non-ND)	Unknown (NOS)
n	408	100	52	49	105	128	36
Age (± SD)	72.7 ± 7.8[a, b]	73.4±6.2[a, b, c]	68.2±7.7[d, e, f]	73.6±4.7[b]	72.0±8.3[b]	63.5±9.0[g]	69.4±10.1
Sex (F/M)	192/216[h,i,j]	30/70 [b,k]	28/24[i]	7/42[b,c,e]	40/65[f]	77/51[c]	13/23
MMSE (± SD)	24.4 ± 4.6[b, h,l,m]	25.9±3.5[b, k, m, n]	24.1±4.9[b,e,m]	23.6±6.4[b,m,o]	26.1±3.8[b,g]	28.0±2.4	27.7±3.6
Education (±SD)	12.6 ± 4.2[p]	11.5±4.0[b]	11.7±3.1[j]	12.6±4.3	11.9±3.8[f]	13.1±3.4	12.8±3.7
Aβ+ (No./Total No., %)	386/394, 98% [q]	37/98, 38%	16/51, 31%	23/49, 47% [j]	42/100, 42%	36/124, 29%	13/32, 41%
Positive tau-PET (No./Total No., %)	304/408, 75% [q]	11/100, 11% [f]	4/52, 8%	8/49, 16% [b]	11/105, 10% [f]	2/128, 2%	3/36, 8%
Tau-PET SUVR, temporal meta-ROI (±SD)	1.77 ± 0.64 [q]	1.21 ± 0.22 [b]	1.20 ± 0.19 [j]	1.25 ± 0.16 [b]	1.22 ± 0.25 [b]	1.13 ± 0.12	1.21 ± 0.23 [f]

[a] p<0.001 vs FTD, [b] p<0.001 vs non-ND, [c] p<0.05 vs NOS, [d] p<0.001 vs DLB, [e] p<0.01 vs ND, [f] p<0.01 vs non-ND, [g] p<0.01 vs NOS, [h] p<0.01 vs VaD, [i] p<0.001 vs DLB, [j] p<0.05 vs non-ND, [k] p<0.01 vs FTD, [l] p<0.001 vs ND, [m] p<0.001 vs NOS, [n] p<0.05 vs DLB, [o] p<0.05 vs ND, [p] p<0.05 vs VaD, [q] p<0.001 vs all other groups.

© 2023 Smith et al. *JAMA Neurology.*

eFigure 1

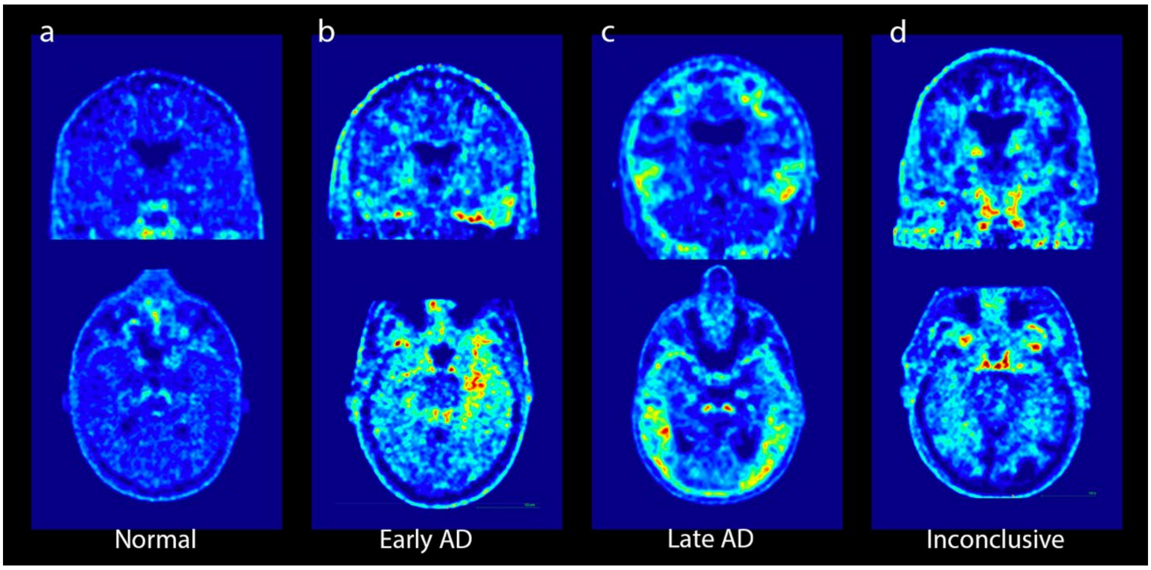

Example images of the visual read categories. Coronal (upper row) and transversal images (bottom row) of a) a normal visual read; b) early AD uptake pattern (temporal lobe uptake); c) late AD uptake pattern; and d) an inconclusive scan. AD = Alzheimer's Disease.

© 2023 Smith et al. *JAMA Neurology.*

eFigure 2

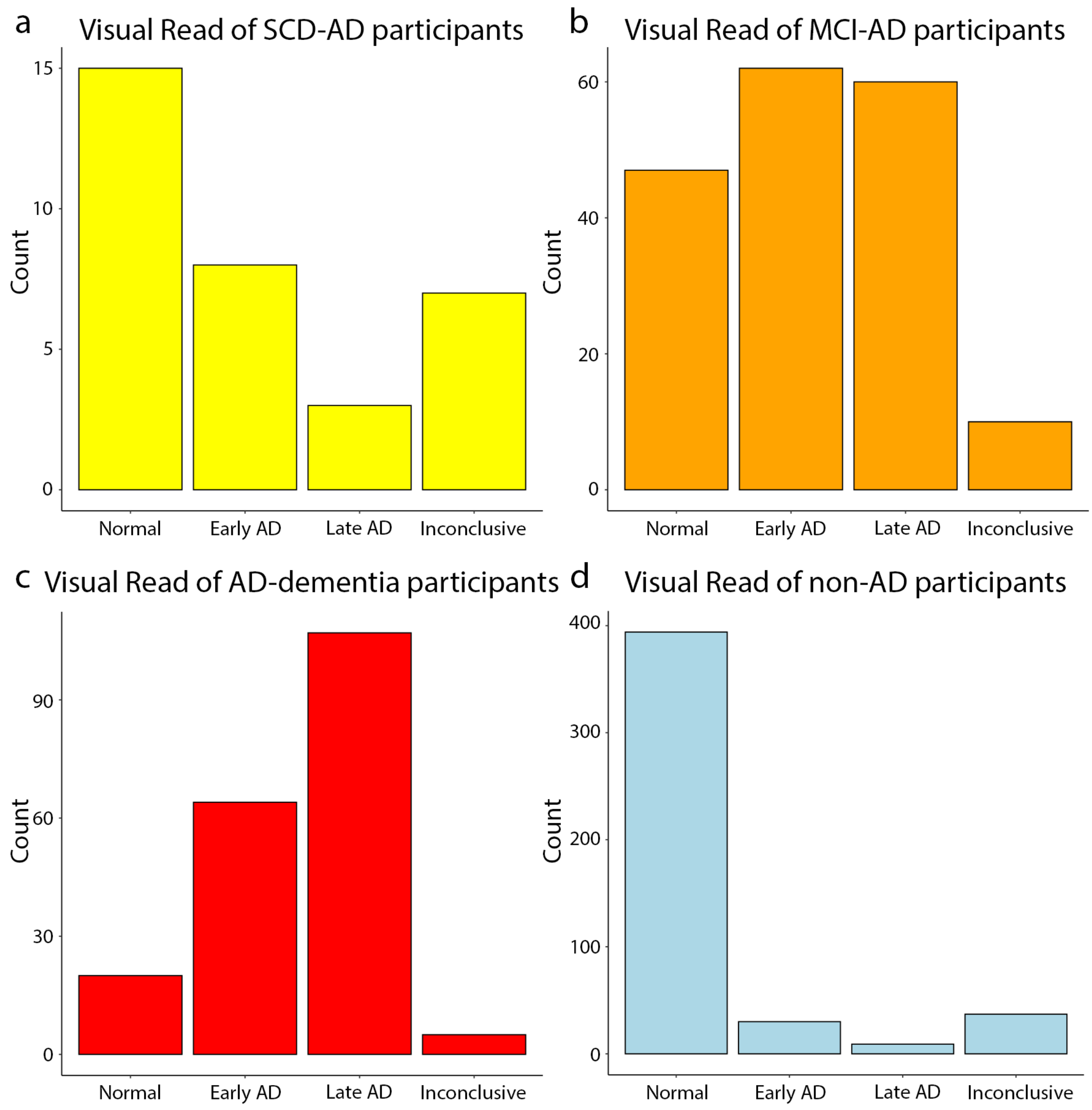

Visual read results in different diagnostic and cognitive groups. The panels show bar graphs of the number of participants within each visual read category for a) participants with SCD with a pre-PET suspected underlying AD; b) participants with a pre-PET MCI-AD; c) participants with a pre-PET AD dementia; and, d) participants with a pre-PET non-AD diagnosis. AD = Alzheimer's Disease, MCI = mild cognitive impairment, SCD = subjective cognitive decline.

© 2023 Smith et al. *JAMA Neurology.*

eFigure 3

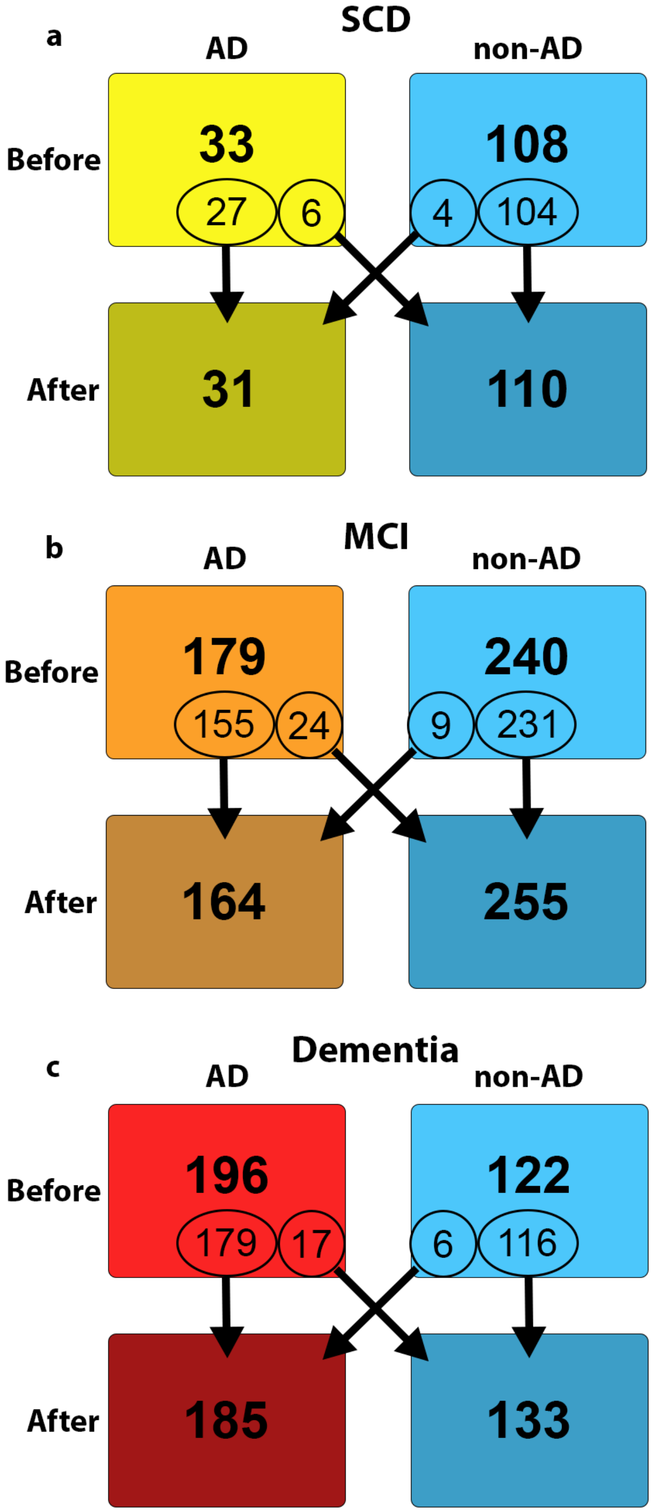

Changes in diagnoses in different cognitive subgroups. Participants changed groups as indicated in a) Subjective cognitive decline, b) Mild cognitive impairment, and c) dementia groups.

© 2023 Smith et al. *JAMA Neurology.*

eFigure 4

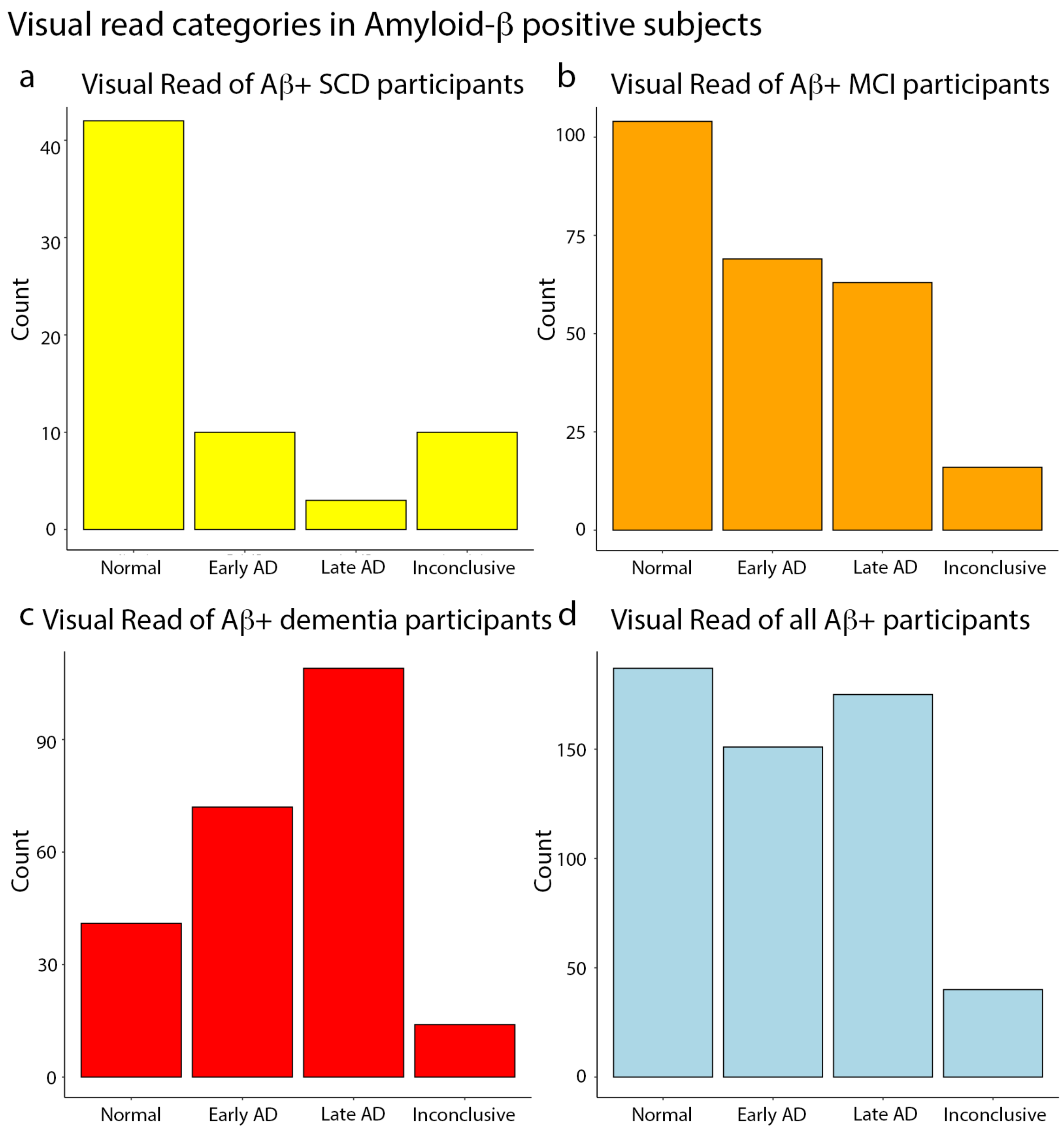

Visual read results in all amyloid-β positives and in amyloid-β positive cognitive subgroups. The panels show bar graphs of the number of participants within each visual read category for amyloid-β positive participants with a) SCD; b) MCI; c) dementia; and, d) all amyloid-β positive participants. Aβ+ = amyloid-β positive, AD = Alzheimer's Disease, MCI = mild cognitive impairment, SCD = subjective cognitive decline.

© 2023 Smith et al. *JAMA Neurology.*

eFigure 5

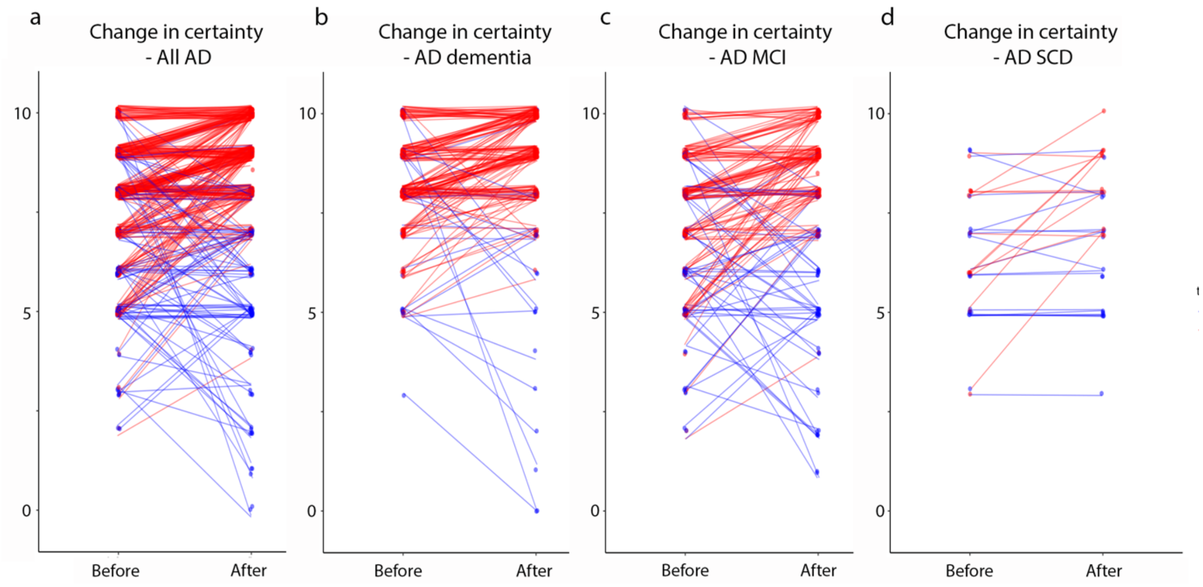

Change in certainty with the added information from the visual read of the [^{18}F]RO948 PET scan in participants with a baseline diagnosis of Alzheimer's Disease (AD). Participants with a positive visual read are depicted in red and participants with a negative visual read in blue. Results for all participants in a), b) to d) shows the participants grouped by cognitive status: b) AD dementia, c) mild cognitive impairment, and d) subjective cognitive decline. A jitter of the datapoints and connecting lines has been introduced to visualize the participants with identical data, in some instances this results in the lines not ending up perfectly in the dots.

© 2023 Smith et al. *JAMA Neurology.*

eFigure 6

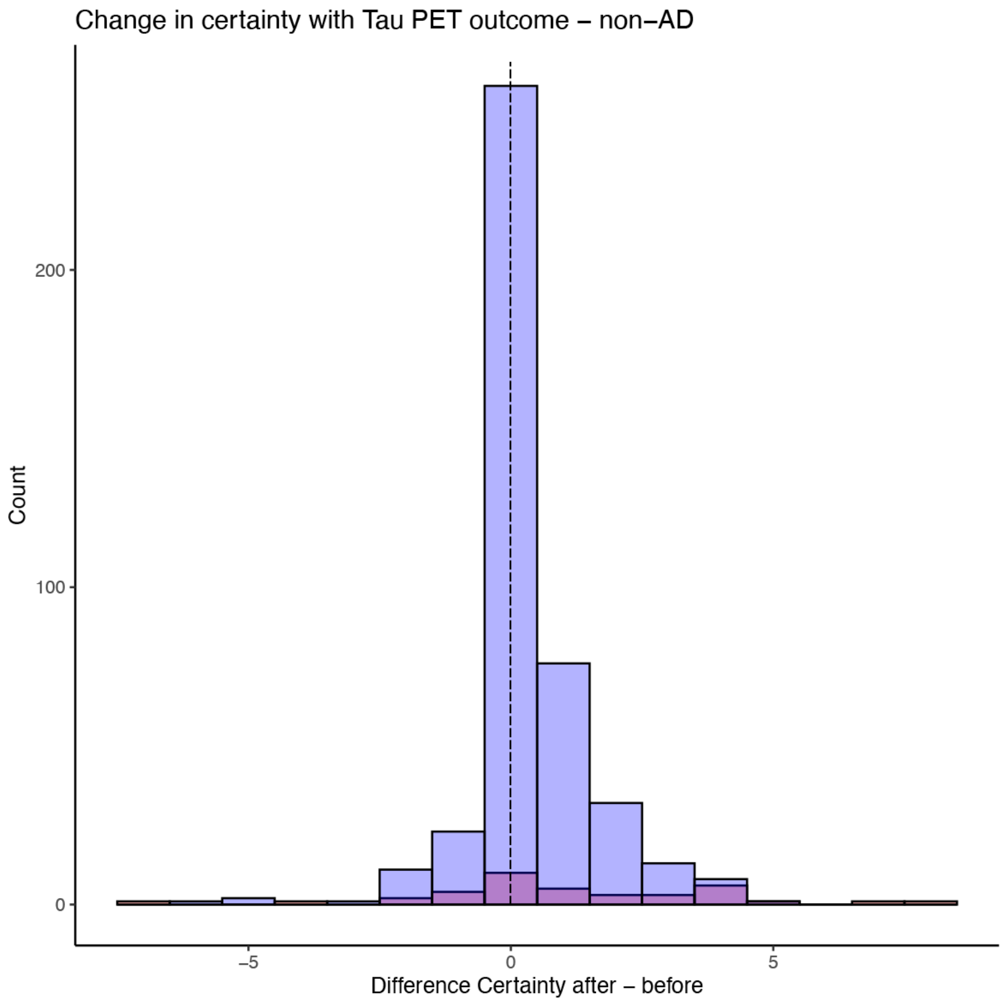

Change in certainty in the group with a non-AD diagnosis at baseline. Negative visual reads are depicted in blue, positive in red. Overlapping blue and red are visualized as purple. Dashed line indicates the zero point (no change).

© 2023 Smith et al. *JAMA Neurology.*

eFigure 7

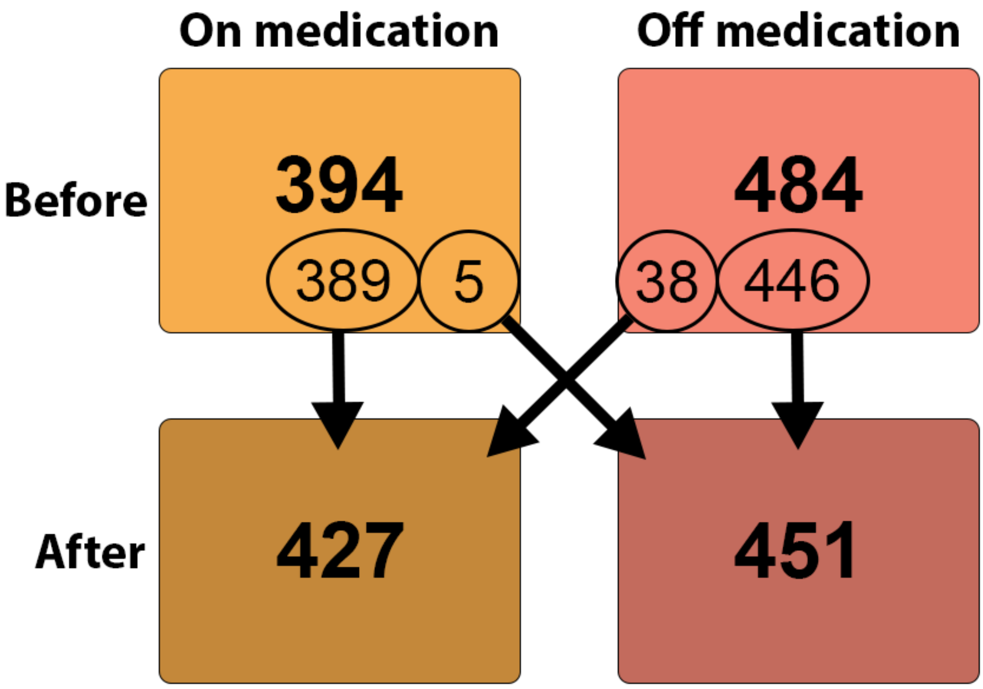

Sensitivity analysis of changes in medication focusing on medication to enhance cognitive functions and not including antidepressant medication. McNemar's test p<0.001. The participants with an increase in medication (addition of a medication with a new mode of action) were considered "off" medication before PET.

© 2023 Smith et al. *JAMA Neurology.*

Translated forms used in the study

PART I: Patient data

Completed by: ……………………………………..…. Date: ……. …../………../…20…………

1. How do you assess the patient's cognitive impairment? (*choose <u>one</u> option*)

- □ Subjective memory impairment
- □ MCI
- □ Dementia

2. How confident are you about your assessment?

Not at all sure |—|—|—|—|—|—|—|—|—|—| Absolutely sure

3. What is the most probable etiology of the patient's cognitive impairment? *(choose <u>one</u> option)*

- □ Alzheimer's disease
- □ Vascular dementia
- □ Frontotemporal dementia
- □ Dementia with Lewy bodies
- □ Other neurodegenerative disease, namely ………………………………………………………
- □ No neurodegenerative disease, namely ………………………………………………………
- □ Unclear

4. How sure are you of the etiology?

Not at all sure |—|—|—|—|—|—|—|—|—|—| Absolutely sure

5. Etiological differential diagnoses? *(choose <u>one or more</u> options)*

- □ Alzheimer's disease
- □ Vascular dementia
- □ Frontotemporal dementia
- □ Dementia with Lewy bodies
- □ Other neurodegenerative disease, namely ………………………………………………………
- □ No neurodegenerative disease, namely ……………………………………………
- □ Unclear

6. Expected result from PET?

□ Tau - PET positive □ Tau-PET negative

© 2023 Smith et al. *JAMA Neurology.*

7. Would you have ordered Tau-PET if the patient had not participated in the study (if this was possible)?

□ Yes □ No

8. Have further examinations/tests been planned to ensure diagnosis*?

□ Yes □ No

If so, list these in chronological order by entering a number on the line before each item with the first examination listed as '1', the second examination as '2' and so on. If two or more examinations were to be carried out at the same time, enter the same number for them.

_____ Clinical follow-up (for diagnosis*), planned in ….. (enter number of years)
_____ Iterated cognitive assessment performed by a nurse (for diagnostic purposes)
_____ Neuropsychologist assessment (for diagnostic purposes)
_____ Occupational therapist assessment (for diagnostic purposes)
_____ New lumbar puncture (for diagnostic purposes)
_____ Laboratory tests, specify: ………………………………………………………………
_____ FDG-PET of the brain
_____ DaTscan-SPECT or FE-PE2I-PET
_____ Speech therapist assessment (for diagnostic purposes)
_____ Second opinion by a neurologist
_____ Second opinion by a psychiatrist
_____ Other, namely ……………………………………………………….……………………

*This means follow-up visit/examination that is done with the main purpose of ensuring a clinical diagnosis, and not mainly for starting treatment/care planning or research.

9. Does the patient have medication for dementia-related or psychiatric symptoms?

□ Yes □ No

If so, briefly specify which drugs (not dose): ……………………..…………

© 2023 Smith et al. *JAMA Neurology.*

PART II: Tau-PET data

Interpreting doctor: Douglas Hägerström / Ruben Smith. PET- Examination date: DD/MM/YYYY

10. Summary assessment

□ Tau -PET pathological □ Tau-PET normal □ Tau-PET inconclusive

11. Description of Tau-PET:

12. Categorization of the findings (see interpretation template below)

□ A
□ B
□ C
□ D

© 2023 Smith et al. *JAMA Neurology.*

Tau-PET interpretation template:

A) If Tau-PET is clearly normal:
- If the patient exhibits cognitive symptoms as well as objective cognitive impairment (i.e. at the level of mild cognitive impairment (MCI) or dementia), then Tau-PET strongly suggests that these symptoms are not caused by Alzheimer's pathology. It is therefore much more likely that other factors or diseases are causing the cognitive impairment. If CSF Ab42/40 (alternatively Ab42) and/or Amyloid-PET are also normal, this speaks even more to the fact that there is no Alzheimer's disease present (not even a preclinical one).
- If CSF Ab42/40 (and possibly Ab42) or Amyloid-PET are pathological, the patient may have a yet asymptomatic (preclinical) Alzheimer's or possibly an Alzheimer's with only mild subjective symptoms. In contrast, a normal Tau-PET indicates that the patient will not develop Alzheimer's dementia within the next few years.

B) If Tau-PET is clearly pathological in temporal regions of the brain (corresponding to Braak stage I-IV):
- If the patient exhibits problems with short-term memory or language function, the CSF Ab42/40 (and possibly Ab42) or Amyloid-PET are also pathological, the Tau-PET examination strongly suggests that Alzheimer's pathology causes all or at least part of the symptomatology. This applies regardless of cognitive level, i.e. mild cognitive impairment (MCI), dementia, but also cases with subjective symptoms (SCD).
- If CSF Ab42/40 (alternatively also Ab42) or Amyloid-PET are normal, a discussion with the responsible researcher at the diagnostic conference is recommended.

C) If the Tau-PET is clearly pathological in temporal as well as parieto-occipital and possibly also frontal regions of the brain (corresponding to Braak stage V-VI):
- If the patient exhibits problems with short-term memory, language function, executive or visuospatial ability and if CSF Ab42/40 (alternatively Ab42) or Amyloid-PET are also pathological, the Tau-PET examination strongly suggests that it is Alzheimer's pathology that causes the majority of symptoms. This applies regardless of cognitive level, i.e. mild cognitive impairment (MCI), dementia, but also cases with subjective symptoms (SCD).
- If CSF Ab42/40 (alternatively also Ab42) or Amyloid-PET are normal, a discussion with the responsible researcher at the diagnostic conference is recommended.

D) If the Tau-PET is in the borderline zone:
- If the patient exhibits subjective cognitive symptoms (SCD) or mild cognitive impairment (MCI) where problems with short-term memory or language function are present, then Tau-PET may be consistent with incipient Alzheimer's pathology. It is then important that CSF Ab42/40 (alternatively Ab42) and/or Amyloid-PET are also pathological. A new clinical examination and Tau-PET are recommended in about 1 year.
- If the patient exhibits a pronounced cognitive impairment and, above all, if the symptomatology is not limited to short-term memory or language function, another diagnosis should be considered.

© 2023 Smith et al. *JAMA Neurology.*

PART III: Patient data

Completed by: ……………………………….….. Date: ……. …../………../…20…………

13. PET assessment?

□ Tau-PET positive □ Tau-PET negative

14. Are the results of the cerebrospinal fluid analysis known?

□ Yes □ No

15. Was the Tau-PET examination helpful in any way? (*choose one option*)

□ My etiological diagnosis of the patient's illness is based to a very large extent on the diagnostic information from the PET scan
□ Tau-PET results have provided information that has increased my understanding of the etiology to the patient's illness significantly
□ Tau-PET results have not had any major impact on my understanding of the patient's disease
□ Tau-PET results got me confused regarding the patient's illness but have not been a reason for ordering further tests or examinations
□ Tau-PET results got me confused regarding the patient's illness and have led to further examination(s) that I would not have ordered otherwise

16. How much help do you think you had from the Tau-PET examination in diagnostics?

None at all |—|—|—|—|—|—|—|—|—|—| Very much

17. How do you assess the patient's cognitive impairment? (*choose one option*)

□ Subjective memory impairment
□ MCI
□ Dementia

18. How confident are you about your assessment?

Not at all sure |—|—|—|—|—|—|—|—|—|—| Absolutely sure

19. What is the most likely etiology of the patient's cognitive impairment? (*choose one option*)

□ Alzheimer's disease
□ Vascular dementia

© 2023 Smith et al. *JAMA Neurology.*

□ Frontotemporal dementia
□ Dementia with Lewy bodies
□ Other neurodegenerative disease, namely …………………………………………………
□ No neurodegenerative disease, namely ……………………………………………..
□ Unclear

20. How sure are you of the etiology?

Not at all sure 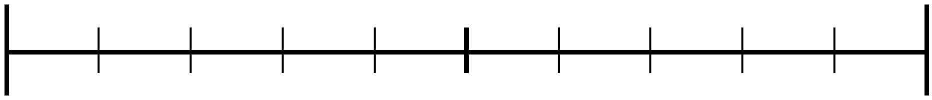 Absolutely sure

21. Has the plan regarding further examination(s) changed due to the PET results or due to the diagnosis/certainty of the diagnosis having changed?

□ Yes □ No

If so, list these in chronological order by entering a number on the line before each item with the first survey listed as '1' and the second survey as '2' and so on. If two or more examinations were to be carried out at the same time, enter the same number for them.

____ Clinical follow-up (for diagnosis*), planned in ….. (enter number of years)
____ Renewed cognitive assessment performed by a nurse (for diagnostic purposes)
____ Neuropsychologist assessment (for diagnostic purposes)
____ Occupational therapist assessment (for diagnostic purposes)
____ New lumbar puncture (for diagnostic purposes)
____ Laboratory tests, specify: ……………………………………………………………
____ FDG -PET of the brain
____ DaTscan-SPECT or FE-PE$_2$ I-PET
____ Speech therapist assessment (for diagnostic purposes)
____ Second opinion by a neurologist
____ Second opinion by a psychiatrist
____ Other, namely ……………………………………………………….…………………

*This means follow-up visit/examination that is done with the main purpose of ensuring a clinical diagnosis, and not mainly for starting treatment/care planning or research.

22. Has the medication for dementia-related or psychiatric symptoms changed because of the PET results or because the certainty of the diagnosis has changed?

□ Yes □ No

If so, briefly specify which drugs (not dose): …………………….………..

© 2023 Smith et al. *JAMA Neurology.*

References

1. Rogasch JM, Suleiman S, Hofheinz F, et al. Reconstructed spatial resolution and contrast recovery with Bayesian penalized likelihood reconstruction (Q.Clear) for FDG-PET compared to time-of-flight (TOF) with point spread function (PSF). *EJNMMI Phys.* 2020;7(1):2.

© 2023 Smith et al. *JAMA Neurology.*
