# Supplementary material for: Clinical Utility of Tau Positron Emission Tomography in the Diagnostic Workup of Patients With Cognitive Symptoms
Source: JAMA Neurol. 2023 May 22;80(7):749–56. doi: 10.1001/jamaneurol.2023.1323 (PMC10203972; doi:10.1001/jamaneurol.2023.1323)
Supplement: Supplement 2. — Data sharing statement [file jamaneurol-e231323-s002.pdf]

Data Sharing Statement

Smith. Clinical Utility of Tau Positron Emission Tomography in the Diagnostic Workup of Patients With Cognitive Symptoms. *JAMA Neurol*. Published May 22, 2023. doi:10.1001/jamaneurol.2023.1323

Data
Data available: Yes
Data types: Other (please specify)
Additional Information: Anonymized data will be shared by request from a qualified academic investigator for the sole purpose of replicating procedures and results presented in the article and as long as data transfer is in agreement with EU legislation on the general data protection regulation and decisions by the Swedish Ethical Review Authority, which should be regulated in a material transfer agreement.
How to access data: oskar.hansson@med.lu.se
When available: With publication

Supporting Documents
Document types: None

Additional Information
Who can access the data: Anonymized data will be shared by request from a qualified academic investigator as long as data transfer is in agreement with EU legislation on the general data protection regulation and decisions by the Swedish Ethical Review Authority, which should be regulated in a material transfer agreement.
Types of analyses: Data will be shared for the sole purpose of replicating procedures and results presented in the article.
Mechanisms of data availability: Data sharing should be regulated in a material transfer agreement.
